# Nutritional Risk Among Emergency Department Patients with Diabetes Mellitus or Chronic Kidney Disease: A Single-Center Prospective Observational Comparative Study

**DOI:** 10.3390/nu18142241

**Published:** 2026-07-09

**Authors:** Sema Ayten, Emine Ünal, Michal Pruc, Iwona Niewiadomska, Lukasz Szarpak

**Affiliations:** 1 Department of Emergency Medicine, İstanbul Göztepe Prof. Dr. Süleyman YALÇIN City Hospital, Istanbul 34722, Turkey; 2 Institute of Medical Science, The John Paul II Catholic University of Lublin, 20-708 Lublin, Poland; michal.pruc@kul.pl (M.P.);; 3 Institute of Psychology, The John Paul II Catholic University of Lublin, 20-950 Lublin, Poland

**Keywords:** diabetes mellitus, chronic kidney disease, malnutrition, nutritional assessment, hospital admission, emergency department disposition

## Abstract

Background/Objectives: Patients with diabetes mellitus (DM) and chronic kidney disease (CKD) frequently present to emergency departments (EDs) with acute metabolic or renal complications. Nutritional risk may coexist with disease severity in this population, but its relationship with hospital admission remains insufficiently characterized. This study aimed to compare the nutritional status between admitted and discharged ED patients with DM and/or CKD. Materials and Methods: This single-center prospective observational comparative study included 101 adult ED patients with documented DM and/or CKD. Nutritional status was assessed during the ED encounter using the Nutritional Risk Screening-2002 (NRS-2002) and Mini Nutritional Assessment (MNA). Clinical, anthropometric, and laboratory variables were compared between admitted and discharged patients. Logistic regression was used to explore factors associated with hospital admission. Results: Of 101 patients, 51 were admitted, and 50 were discharged. NRS-2002-defined nutritional risk was more frequent among admitted than discharged patients (64.7% vs. 30.0%; risk difference: 34.7 percentage points, 95% CI: 16.4–53.0; *p* = 0.001). MNA-defined malnutrition was also more frequent among admitted patients (45.1% vs. 12.0%; risk difference for malnutrition alone: 33.1 percentage points, 95% CI: 16.7–49.5), and the overall MNA category distribution differed significantly between groups (*p* < 0.001). Admitted patients had higher C-reactive protein and urea concentrations and lower lymphocyte counts (*p* = 0.001 for all), while creatinine showed a borderline between-group difference (*p* = 0.049). In the exploratory adjusted model, hospital admission was associated with serum urea (OR: 1.018, 95% CI: 1.007–1.029; *p* = 0.001), CRP (OR: 1.022, 95% CI: 1.008–1.036; *p* = 0.002), female sex (OR: 0.307, 95% CI: 0.098–0.965; *p* = 0.043), and MNA-defined malnutrition compared with normal nutritional status (OR: 7.926, 95% CI: 1.482–42.391; *p* = 0.016), although estimates were limited by the number of admission events. Conclusion: Among ED patients with DM and/or CKD, nutritional risk was substantially more common in those requiring hospital admission. This association likely reflects confounding by indication and possible reverse causation, as disposition decisions may incorporate frailty, general appearance, functional decline, and clinical vulnerability, which are also partly captured by nutritional screening tools. Nutritional screening should therefore be interpreted as a marker of clinical vulnerability rather than as an isolated determinant of disposition.

## 1. Introduction

Malnutrition and nutritional risk are common among patients with chronic diseases and are associated with adverse clinical outcomes, including increased morbidity, mortality, and health-care utilization [1,2]. In the emergency department (ED), patients with diabetes mellitus (DM) and chronic kidney disease (CKD) frequently present with acute metabolic, infectious, or renal complications, and disposition decisions must often be made under time constraints. In this setting, nutritional risk is unlikely to represent a purely dietary construct. In patients with DM and/or CKD, acute infection, dehydration, uremia, metabolic acidosis, hyperglycemic crises, and reduced oral intake may increase catabolic stress and accelerate loss of functional reserve. At the same time, renal dysfunction and systemic inflammation may independently affect appetite, muscle mass, immune response, and physical performance. Therefore, a positive nutritional screening result in the ED may capture the cumulative burden of chronic disease, acute physiological stress, frailty, and metabolic instability rather than malnutrition alone. This interpretation is consistent with the GLIM framework, which conceptualizes malnutrition as the result of phenotypic criteria combined with etiologic drivers such as reduced intake, inflammation, and disease burden [3,4]. This overlap is particularly important when nutritional screening findings are interpreted in relation to hospital admission, because the same processes that worsen nutritional scores may also contribute to the clinical decision to admit the patient [5,6,7,8].

Unlike inpatient wards, the ED provides only a limited time window for clinical assessment, and nutritional status is rarely the primary focus during acute evaluation. Nevertheless, many ED patients with chronic diseases present with reduced oral intake, dehydration, infection, metabolic instability, or functional decline, all of which may interact with nutritional vulnerability [9,10]. Therefore, identifying nutritional risk in the ED may have clinical value not as a standalone diagnostic or disposition criterion, but as part of a broader assessment of clinical vulnerability.

DM is characterized by chronic hyperglycemia and impaired carbohydrate, lipid, and protein metabolism [11,12]. Several mechanisms may contribute to impaired nutritional status in patients with DM. Poor glycemic control may promote osmotic diuresis, dehydration, metabolic instability, and increased protein catabolism, particularly during acute illness. In addition, insulin resistance and inflammatory activation may contribute to loss of muscle mass and reduced functional reserve, even in patients with preserved or elevated body mass index. Diabetes-related gastrointestinal symptoms, including gastroparesis, may reduce oral intake, while dietary restrictions, medication-related adverse effects, recurrent infections, reduced physical activity, and multimorbidity may further compromise nutritional status [13,14,15]. In the ED setting, these mechanisms may become clinically apparent during acute metabolic decompensation, infection, dehydration, or functional decline. Therefore, nutritional risk in patients with DM may reflect both chronic metabolic vulnerability and the physiological stress of the acute presentation.

CKD is defined by persistent kidney damage or reduced kidney function and may progress to kidney failure over time [16]. As renal function declines, patients may develop uremic symptoms, inflammation, metabolic disturbances, reduced appetite, and protein-energy wasting [17,18,19]. In addition, dietary restrictions involving fluid, sodium, potassium, phosphorus, and protein intake may further increase the risk of nutritional deficiencies and adverse clinical outcomes [17,19,20]. The overlap between DM and CKD is particularly relevant because both conditions may amplify catabolic and inflammatory pathways. Hyperglycemia, insulin resistance, uremia, metabolic acidosis, dietary limitations, and recurrent infections may contribute to reduced muscle mass, impaired immune response, and decreased functional reserve. Consequently, nutritional risk in this population may be difficult to separate from underlying disease severity [21,22].

Although malnutrition and nutritional risk have been extensively evaluated in hospitalized patients, dialysis populations, geriatric cohorts, and disease-specific groups after hospital admission, fewer data are available at the point of ED presentation. This represents an important evidence gap because the ED is often the first point of acute care, where clinicians must rapidly distinguish patients who require inpatient treatment from those who can be safely discharged. In patients with DM and/or CKD, this distinction may be particularly complex because nutritional risk may coexist with acute kidney injury, uremia, infection, dehydration, hyperglycemic crises, inflammation, and functional decline. Therefore, comparing admitted and discharged patients may help clarify whether impaired nutritional status is concentrated among patients with greater acute clinical severity or whether a meaningful burden of nutritional vulnerability is also present among those discharged from the ED. The latter is clinically relevant because discharged patients may not receive inpatient nutritional assessment, despite having chronic disease-related dietary limitations, reduced intake, or early functional deterioration that may affect subsequent outcomes [23,24]. Thus, characterizing nutritional risk at the time of ED presentation may provide information about clinical vulnerability across the full spectrum of ED disposition, rather than only after hospital admission.

The Nutritional Risk Screening-2002 (NRS-2002) is widely used for nutritional risk screening in adult hospital populations, whereas the Mini Nutritional Assessment (MNA) is primarily established in older adults [25]. Therefore, the use of both instruments may provide complementary information, although MNA-based findings should be interpreted cautiously in a broader adult ED population. However, an important gap remains in the literature: nutritional risk has not been sufficiently characterized at the time of ED presentation among patients with DM and/or CKD, particularly in relation to whether patients are admitted to hospital or discharged after acute evaluation. Moreover, it remains unclear whether nutritional screening findings in this population reflect an independent nutritional burden or whether they mainly overlap with renal dysfunction, inflammation, metabolic instability, and acute illness severity. Addressing this gap is important because ED-based nutritional screening could help identify clinically vulnerable patients across the full spectrum of ED disposition, including those who are discharged without inpatient nutritional assessment.

This study aimed to compare nutritional risk between admitted and discharged ED patients with documented DM and/or CKD and to explore whether nutritional screening findings were associated with hospital admission after accounting for available markers of renal, inflammatory, and metabolic severity. The study was not designed to develop or validate a prediction model for ED disposition.

## 2. Materials and Methods

### 2.1. Study Design and Setting

This was a single-center prospective observational comparative study conducted in the adult ED of a tertiary state hospital in Istanbul between 15 July 2024 and 15 October 2024. The study was reported in accordance with the Strengthening the Reporting of Observational Studies in Epidemiology (STROBE) recommendations for observational studies [26].

### 2.2. Study Population

Eligible patients were adults aged 18 years or older who presented to the ED during the study period and had a documented diagnosis of DM and/or CKD. The presence of DM and CKD was determined from patient history and confirmed using the national electronic health record system and hospital medical records. The admitted group consisted of eligible patients who were admitted to the hospital after ED evaluation. The discharged group consisted of eligible patients who were evaluated in the ED and discharged without hospital admission. Discharged patients did not require inpatient treatment at the time of the index ED visit.

The primary outcome was hospital admission from the ED. Nutritional status was evaluated using two screening instruments: the Nutritional Risk Screening-2002 (NRS-2002) [27] and the Mini Nutritional Assessment (MNA) [28]. NRS-2002-defined nutritional risk, defined as a score ≥3, was considered the main adult-oriented nutritional screening measure for descriptive group comparisons and crude association analyses because it is widely used in adult hospital populations. Because NRS-2002 incorporates disease severity and acute clinical stress, it was not used as the nutritional covariate in the available adjusted model that also included markers of renal and inflammatory severity. MNA-defined malnutrition was evaluated as a complementary nutritional assessment measure and was used as the available nutritional-status variable in the exploratory adjusted model, with cautious interpretation because the MNA was originally developed and validated mainly for older adults. Nutritional assessments were performed during the ED encounter after the initial clinical evaluation. In admitted patients, assessments were performed after the diagnosis of the acute condition had been established and around the time of the admission decision. In discharged patients, assessments were performed before discharge from the ED. Because nutritional assessment was performed during the ED encounter and around the disposition decision, the study evaluated associations with hospital admission status rather than prospective prediction of admission before disposition. Age and sex were recorded from the hospital electronic medical record system. Body weight and height were obtained from patient self-report during the ED encounter or from the most recent available hospital record when direct measurement was not feasible. Body mass index was calculated as weight in kilograms divided by height in meters squared. Nutritional assessment results, hospital admission status, admission ward, and routine laboratory parameters were also recorded. Age, sex, and body mass index were recorded because of their potential association with nutritional status. Laboratory parameters obtained at ED presentation were recorded because they may reflect nutritional, inflammatory, renal, and metabolic status.

For admitted patients, the admitting ward and the main reason for hospitalization were recorded from the hospital information system. Demographic and anthropometric data were collected prospectively during the ED encounter. Based on these data, the nutritional status of patients admitted to the internal medicine ward or the intensive care unit was compared. Patients with a total NRS-2002 score of ≥3 were classified as being at nutritional risk, according to the established NRS-2002 scoring criteria [27]. According to the MNA, patients with scores of 24–30 were classified as having normal nutritional status, patients with scores of 17–23.5 were classified as being at risk of malnutrition, and patients with scores below 17 were classified as malnourished [28]. Patients were classified separately according to the predefined categories of each nutritional assessment tool. When the two instruments yielded conflicting results, patients were classified according to the result of the relevant scale being analyzed. Therefore, no reclassification or prioritization of one instrument over the other was performed. This approach was chosen to avoid misclassification and to allow for comparison of the clinical association of each nutritional assessment instrument with hospital admission.

Blood samples obtained from the study participants were collected at the time of initial presentation to the ED and consisted of routinely requested laboratory parameters. The variables recorded for the present analysis were age, sex, height, weight, body mass index, nutritional assessment results, hospital admission status, ward of admission among admitted patients, and available routine laboratory parameters, including hemoglobin, hematocrit, lymphocyte count, C-reactive protein, glucose, urea, creatinine, calcium, albumin, and total protein.

### 2.3. Exclusion Criteria

Patients were excluded if they were younger than 18 years, pregnant, presented with trauma, had a history of malignancy, had no documented history of DM or CKD, had missing data for the primary exposure or primary outcome, or did not provide voluntary informed consent. Acute infectious or inflammatory conditions at ED presentation were not exclusion criteria, because such conditions are common reasons for ED evaluation in patients with DM and/or CKD and may contribute to the real-world clinical severity captured in this study. Known chronic inflammatory or immune-mediated diseases were not predefined exclusion criteria and were not systematically excluded unless they overlapped with another exclusion criterion, such as malignancy.

### 2.4. Missing Data

The number and proportion of missing values were assessed for all key study variables. Patients with missing data for the primary exposure or primary outcome were excluded. Complete-case analysis was used for the primary analysis. When missingness was present for secondary covariates, the extent of missing data was reported and considered in the interpretation of the findings.

### 2.5. Timing of Nutritional Assessment

Nutritional assessments were performed during the index ED encounter after initial stabilization and routine clinical evaluation. In admitted patients, screening was performed after the acute diagnosis had been established and around the time of the admission decision; in discharged patients, screening was performed before discharge. Therefore, nutritional scores were analyzed in relation to final emergency department disposition and were not interpreted as pre-disposition predictive tests.

### 2.6. Ethical Approval and Consent Forms

Ethical approval for the study was obtained from the Istanbul Medipol University Non-Interventional Clinical Research Ethics Committee with the electronic code 580FDFF4XC. The study was conducted at İstanbul Göztepe Prof. Dr. Süleyman YALÇIN City Hospital. At the time of ethics submission, the local ethics committee of the study site was undergoing institutional renewal and was not accepting new applications; therefore, ethics review was sought from the Istanbul Medipol University Non-Interventional Clinical Research Ethics Committee as an available authorized ethics committee. The study was conducted in accordance with the Declaration of Helsinki, and signed informed consent was obtained from all patients included in the study.

### 2.7. Statistical Analysis

The normality of continuous variables was assessed using the Shapiro–Wilk test. Continuous variables were summarized as mean with standard deviation or median with interquartile range, depending on the distribution. Categorical variables were summarized as counts and percentages. For comparisons of continuous variables between two independent groups, the independent samples *t*-test was used for normally distributed variables, whereas the Mann–Whitney U test was used for non-normally distributed variables. For comparisons of continuous variables across more than two groups, one-way analysis of variance (ANOVA) was used for normally distributed variables, whereas the Kruskal–Wallis test was used for non-normally distributed variables. Between-group differences were reported using absolute mean or median differences and risk differences with 95% confidence intervals where appropriate.

The primary analysis evaluated the association between nutritional risk and hospital admission. Crude odds ratios with 95% confidence intervals were calculated. Multivariable logistic regression was then used to explore whether nutritional risk remained associated with admission after adjustment for prespecified clinically relevant covariates. Overall model performance was assessed using the omnibus model chi-square and Nagelkerke R^2^. This model was used to estimate adjusted associations and was not intended for clinical prediction or disposition decision-making. Because of the limited sample size, the available exploratory adjusted model was restricted to a small number of covariates selected a priori: age, sex, serum urea as the available renal and metabolic severity marker, C-reactive protein (CRP) as the inflammatory marker, and MNA-defined nutritional status, entered as a three-category variable with normal nutritional status as the reference category, as the available nutritional-status variable. NRS-2002 and MNA were not entered simultaneously into the same multivariable model because of conceptual overlap between the instruments. In addition, NRS-2002 includes a disease-severity component, which may overlap with markers of acute renal and inflammatory severity, including serum urea and CRP. Therefore, an adjusted model including NRS-2002 together with these severity markers was not used as a primary adjusted analysis, because such a model would risk partial overadjustment and would be difficult to interpret biologically. Instead, NRS-2002 was retained as the main adult-oriented screening tool for descriptive comparisons, crude association analysis, and exploratory ROC analysis. Therefore, MNA-defined nutritional status was used as the nutritional-status variable in the available exploratory adjusted model. Multivariable findings were interpreted cautiously because of the limited sample size, the small number of outcome events, and the primarily geriatric validation context of the MNA. An age-restricted multivariable sensitivity analysis among patients aged ≥65 years was considered clinically relevant but could not be reliably performed because of the limited sample size. However, to address the age-specific applicability of the MNA, a descriptive post hoc comparison of MNA categories was performed between patients aged <65 years and those aged ≥65 years using the Pearson chi-square test. This analysis was considered exploratory and underpowered and was not intended to validate MNA performance in either age subgroup. Agreement between NRS-2002 and MNA classifications was assessed using Cohen’s kappa after clinically appropriate dichotomization. Univariable receiver operating characteristic (ROC) analyses were performed exploratorily and separately for selected variables to describe how nutritional scores and laboratory markers separated admitted from discharged patients. Area under the curve (AUC) values were reported descriptively with 95% confidence intervals. No formal statistical comparisons between AUC values were performed, and small numerical differences between AUC estimates were not interpreted as evidence of superiority of one marker over another. Because these ROC analyses were exploratory, AUC values and their 95% confidence intervals were presented descriptively without formal pairwise inferential comparison. These analyses were exploratory and were not intended to develop, validate, or recommend a prediction model for hospital admission.

Given the exploratory nature of the study and the fixed sample size, no formal prospective sample size calculation was performed. Instead, effect estimates were presented with 95% confidence intervals to reflect statistical precision. A two-sided *p* value below 0.05 was considered statistically significant. Analyses were performed using SPSS version 23.

## 3. Results

A total of 101 patients were included in the study, comprising 51 hospitalized patients and 50 discharged patients. Of the patients, 48 (47.5%) were female, and 53 (52.5%) were male, with a mean age of 67.1 ± 18.8 (21–104) years. Additionally, there were 30 (60%) males in the discharged group and 23 (45.1%) males in the admitted group, and the distribution of genders across groups was similar (*p* = 0.134). Of the admitted patients, 46 (90.2%) were hospitalized in internal medicine wards, and 5 (9.8%) were admitted to the intensive care unit. Data for the primary exposure and primary outcome were complete. CRP and total protein values were missing in one admitted patient each; all other routinely reported laboratory variables were available for the full study population.

According to the NRS-2002 score, nutritional risk was identified in 33 (64.7%) of the 51 patients in the admitted group and in 15 (30.0%) of the 50 patients in the discharged group. No nutritional risk was identified in 18 (35.3%) patients in the admitted group and in 35 (70.0%) patients in the discharged group. The rate of NRS-2002-defined nutritional risk was significantly higher in the admitted group than in the discharged group, with a risk difference of 34.7 percentage points (95% CI: 16.4–53.0; *p* = 0.001).

According to the MNA scoring system, among the 51 patients in the admitted group, 23 (45.1%) had malnutrition, 22 (43.1%) were at risk of malnutrition, and 6 (11.8%) had normal nutritional status. Among the 50 patients in the discharged group, 6 (12%) had malnutrition, 29 (58%) were at risk of malnutrition, and 15 (30%) had normal nutritional status. The distribution of MNA categories differed significantly between admitted and discharged patients (*p* < 0.001). For MNA-defined malnutrition specifically, the risk difference between admitted and discharged patients was 33.1 percentage points (95% CI: 16.7–49.5).

**Figure 1:**
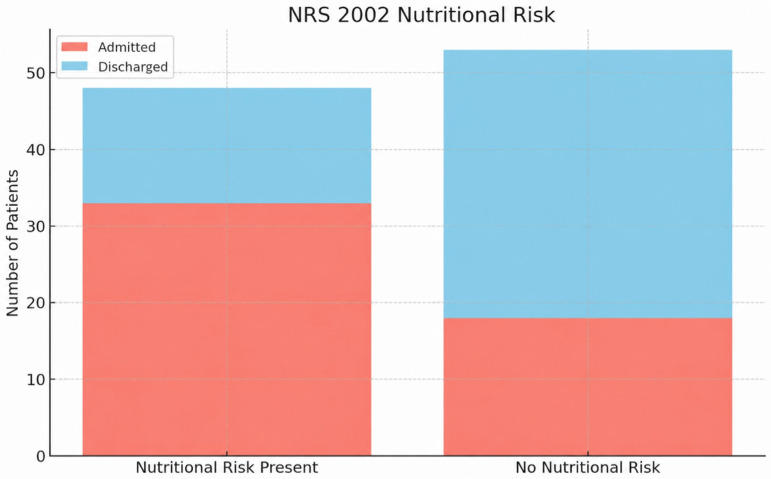
Distribution of nutritional risk according to NRS-2002 among Admitted and discharged patients.

**Figure 2:**
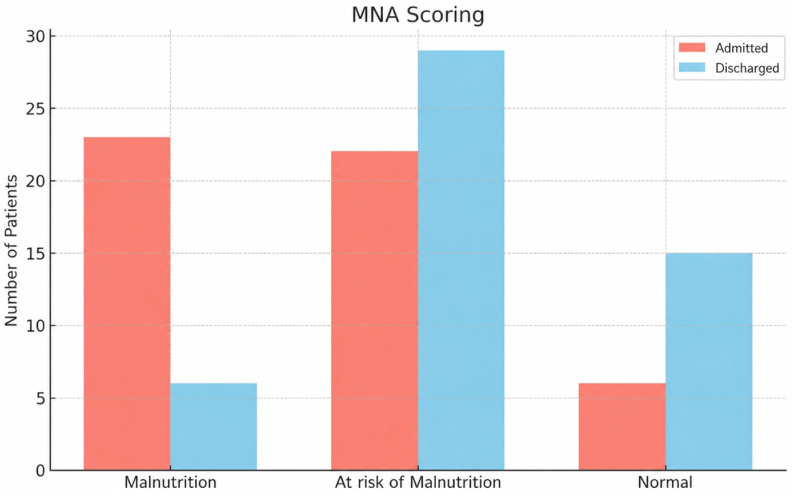
Distribution of nutritional status according to MNA among admitted and discharged patients.

According to both nutritional assessment instruments, impaired nutritional status was more frequent among admitted patients. This difference was significant for NRS-2002-defined nutritional risk (*p* = 0.001) and for the overall MNA category distribution (*p* < 0.001). The distribution of nutritional status according to NRS-2002 and MNA is shown in Figure 1 and Figure 2, respectively.

In crude analysis, MNA-defined malnutrition was associated with hospital admission (OR: 6.02, 95% CI: 2.18–16.63). NRS-2002-defined nutritional risk was also associated with admission (OR: 4.28, 95% CI: 1.86–9.85).

When NRS-2002-defined nutritional risk was evaluated by sex, no significant difference was found between sexes, but according to the MNA score evaluation, normal nutrition was significantly higher in males, and malnutrition was significantly higher in females (*p* = 0.014). However, those at risk of malnutrition were found to be similar in both genders (Table 1).

When admitted and discharged groups were compared in terms of laboratory values, CRP and urea levels were significantly higher in the admitted group, whereas lymphocyte counts were significantly lower. Creatinine levels were also higher in the admitted group, but this difference was of borderline statistical significance (*p* = 0.049). No statistically significant differences were observed for the remaining laboratory parameters (Table 2).

According to the NRS-2002 classification, nutritional risk was identified in 48 (47.5%) individuals, whereas 53 (52.5%) individuals were not classified as being at nutritional risk. Furthermore, according to the classification based on the MNA score, 21 patients (20.8%) had normal nutritional status, 51 patients (50.5%) were at risk of malnutrition, and 29 patients (28.7%) were classified as malnourished. Because the MNA was originally developed and validated mainly for older adults, MNA categories were also examined descriptively according to age group. Of the 101 patients, 33 were aged <65 years and 68 were aged ≥65 years. Among patients aged <65 years, 11 (33.3%) had normal nutritional status, 15 (45.5%) were at risk of malnutrition, and 7 (21.2%) were malnourished. Among patients aged ≥65 years, 10 (14.7%) had normal nutritional status, 36 (52.9%) were at risk of malnutrition, and 22 (32.4%) were malnourished (Table 3). Although older patients showed a numerically higher burden of impaired MNA status, the distribution did not differ significantly between age groups (*p* = 0.086). This age-stratified comparison was descriptive and should be interpreted cautiously because of the limited subgroup sizes.

Age, height, weight, and body mass index were compared between patients with and without NRS-2002-defined nutritional risk separately in the admitted and discharged groups. These subgroup comparisons are presented in Table 4. Similar subgroup comparisons according to MNA nutritional categories are presented in Table 5.

Of the 48 individuals with nutritional risk according to NRS-2002, 24 were classified as malnourished according to the MNA score, 22 were at risk of malnutrition, and the remaining 2 had normal nutritional status. Agreement between NRS-2002 and MNA classifications was limited. When MNA malnutrition or risk of malnutrition was dichotomized against normal nutritional status, Cohen’s kappa was approximately 0.31, indicating fair agreement. This limited agreement supports the decision to analyze the two instruments separately rather than combining them into a single nutritional status variable. Of the 53 individuals without nutritional risk according to NRS-2002, 5 were classified as malnourished according to MNA, 29 were at risk of malnutrition, and 19 had normal nutritional status.

Among admitted patients, NRS-2002-defined nutritional risk was present in 28 of 46 patients admitted to internal medicine wards (60.9%) and in all 5 patients admitted to the intensive care unit (100%). Because the ICU subgroup was very small, this finding should be interpreted descriptively and should not be considered evidence of a statistically robust association.

Among admitted patients, MNA categories differed descriptively between internal medicine and intensive care admissions, although the ICU subgroup was very small. The distribution of admission wards and diagnoses is shown in Table 6.

In exploratory univariable ROC analyses, lymphocyte count showed the highest descriptive discriminatory performance for hospital admission, with an AUC of 0.820 (95% CI: 0.721–0.920; *p* < 0.001). NRS-2002, CRP, MNA, and serum urea showed moderate descriptive separation between admitted and discharged patients, with AUC values of 0.733, 0.726, 0.722, and 0.709, respectively. Creatinine showed limited discriminatory performance, whereas age did not discriminate between admission status. The corresponding 95% confidence intervals are presented in Table 7. The overlapping confidence intervals for NRS-2002, MNA, CRP, and urea support the interpretation that small numerical differences between these AUC estimates should not be interpreted as evidence of superior discriminatory performance of one marker over another. Because these ROC analyses were exploratory and univariable, no formal comparisons between AUC values were performed, and no clinical cut-off values were proposed. For MNA, lower scores indicate poorer nutritional status; therefore, the direction of the association was interpreted accordingly.

In the exploratory multivariable logistic regression model including age, sex, CRP, serum urea, and MNA-defined nutritional status, the overall model was statistically significant (omnibus model chi-square = 50.5, *p* < 0.001), with a Nagelkerke R^2^ of 0.525. The model included 51 admission events and five conceptual predictors, corresponding to approximately 10.2 events per predictor. MNA-defined malnutrition was present in 29 patients overall, including 23 admitted patients and 6 discharged patients. Because MNA nutritional status was entered as a three-category variable, the model included six estimated predictor parameters, corresponding to approximately 8.5 events per parameter. Therefore, the adjusted estimates were interpreted cautiously. In this model, serum urea, CRP, female sex, and MNA-defined malnutrition compared with normal nutritional status were associated with hospital admission, whereas age and MNA-defined risk of malnutrition compared with normal nutritional status were not associated with admission. The wide confidence interval for MNA-defined malnutrition indicates limited precision and supports cautious interpretation of this exploratory model. The results of the multivariable logistic regression model are shown in Table 8.

## 4. Discussion

In this single-center prospective observational study, nutritional risk was more frequent among ED patients with DM and/or CKD who were admitted than among those who were discharged. In crude analyses, both NRS-2002-defined nutritional risk and MNA-defined malnutrition were associated with hospital admission, whereas the adjusted model suggested that nutritional vulnerability clustered with renal dysfunction, inflammatory burden, sex, and acute clinical severity. Given the limited sample size, the lower-bound events-per-parameter ratio, and the wide confidence interval for MNA-defined malnutrition, the adjusted findings should be regarded as hypothesis-generating rather than confirmatory. Overall, the results suggest that nutritional screening captures a broader vulnerability phenotype characterized by impaired intake, uremia, inflammation, frailty, functional decline, and reduced physiological reserve. Therefore, nutritional screening should be interpreted as part of a broader clinical assessment rather than as a stand-alone predictor of ED disposition. The observation that all ICU-admitted patients were nutritionally at risk by NRS-2002 is consistent with clustering of nutritional vulnerability and acute severity, although the very small ICU subgroup precludes separate inference regarding intensive care admission.

The present findings are consistent with previous evidence showing that patients with CKD are particularly vulnerable to protein-energy wasting, dietary inadequacy, inflammation, and adverse clinical outcomes [17,18,19,20,29,30]. In our cohort, higher urea levels and borderline higher creatinine levels among admitted patients support the interpretation that renal and metabolic severity contributed substantially to the admission decision. The finding that serum urea remained associated with admission after adjustment further supports the interpretation that nutritional risk may overlap with renal and metabolic severity rather than represent an isolated factor separating admitted from discharged patients. This interpretation is particularly relevant in patients with CKD because uremia may influence both clinical severity and nutritional status. Reduced appetite, nausea, dietary restrictions, metabolic acidosis, inflammation, and protein-energy wasting may coexist as renal function deteriorates, which is consistent with ESPEN guidance for patients with acute or chronic kidney disease and with the GLIM concept that inflammation and disease burden may act as etiologic drivers of malnutrition [3,4,17,18,19,20,29,30,31]. In an ED setting, elevated urea may therefore represent more than an isolated biochemical abnormality; it may reflect a broader state of metabolic instability and reduced physiological reserve. Consequently, the persistence of urea as an associated factor in the adjusted model supports the concept that nutritional risk and renal severity are closely interconnected in this population, while avoiding overinterpretation of the model as confirmatory.

Similarly, DM may contribute to nutritional vulnerability through poor glycemic control, increased protein catabolism, gastroparesis, medication-related factors, reduced physical activity, and comorbid illness [13,14,15]. The higher age observed among patients with NRS-2002-defined nutritional risk in both admitted and discharged groups is also consistent with previous studies reporting greater malnutrition risk among older patients with chronic disease [2,15,32,33]. Therefore, the interaction between age, DM, CKD, inflammation, and renal dysfunction may explain why nutritional risk was more common among admitted patients in the present study.

The laboratory profile of admitted patients further supports the interpretation that nutritional risk was intertwined with acute illness severity. Higher CRP levels suggest greater inflammatory burden, higher urea and creatinine levels reflect renal and metabolic derangement, and lower lymphocyte counts may indicate systemic stress or impaired immune reserve. This pattern is consistent with previous evidence showing that inflammation, renal dysfunction, and malnutrition frequently coexist and may reinforce each other through reduced intake, catabolic stress, and infection-related vulnerability [17,18,19,32]. The absence of a significant difference in albumin and total protein also suggests that conventional laboratory markers alone may not fully capture nutritional vulnerability in this heterogeneous ED population [34].

The lack of a significant difference in BMI between patients with and without NRS-2002-defined nutritional risk indicates that body mass index alone may be insufficient to identify nutritional vulnerability in this ED population. This is clinically important because patients with chronic metabolic or renal disease may have preserved or elevated BMI despite reduced intake, inflammation, sarcopenia, or functional decline [7,11,13]. In contrast, MNA categories showed a clearer gradient in weight and BMI, particularly among admitted patients, which is consistent with previous evidence showing lower BMI among patients with poorer nutritional status [13]. This pattern is also expected because anthropometric and functional components contribute to MNA classification, whereas NRS-2002 captures nutritional risk in combination with disease severity and acute clinical stress [34].

The limited agreement between NRS-2002 and MNA classifications is an important finding of this study. Although both tools identified a higher burden of impaired nutritional status among admitted patients, they likely captured partially different dimensions of nutritional vulnerability. NRS-2002 incorporates disease severity and acute metabolic stress, which may make it particularly responsive to the acute clinical context of the ED. In contrast, MNA may be more sensitive to chronic nutritional decline, functional impairment, and early nutritional vulnerability, especially in older adults [34].

This distinction is consistent with the observed classification pattern in the present study. NRS-2002 identified nutritional risk in 47.5% of the cohort, whereas MNA classified 28.7% as malnourished and 50.5% as being at risk of malnutrition. Therefore, NRS-2002 may be more suitable as the main adult-oriented screening tool in an acute hospital setting, while MNA may provide complementary information, particularly in older patients. However, because MNA was developed primarily for geriatric populations, MNA-based findings in this broader adult ED cohort should be interpreted cautiously. This concern is particularly relevant because 33 patients in the present cohort were younger than 65 years. In the descriptive age-stratified analysis, impaired MNA status was numerically more frequent among patients aged ≥65 years, but the difference between age groups did not reach statistical significance. Therefore, the MNA-based adjusted findings should be interpreted as exploratory, especially because the study was not powered to assess age-specific MNA performance or to fit a reliable age-restricted adjusted model. The fair agreement between the two tools supports the decision to analyze them separately rather than combine them into a single nutritional variable.

The findings also have implications for patients discharged from the ED. Although admitted patients had a higher frequency of nutritional risk and malnutrition, a clinically relevant proportion of discharged patients also showed impaired nutritional status according to both screening tools. This suggests that discharge from the ED should not necessarily be interpreted as absence of nutritional vulnerability. Some discharged patients may have chronic nutritional risk, early functional decline, or disease-related dietary limitations that do not require immediate hospitalization but may still warrant follow-up. In this context, nutritional screening may help identify patients who could benefit from outpatient nutritional assessment, primary care follow-up, nephrology or diabetes care review, or dietetic counseling after the acute ED episode [2].

From a clinical perspective, these findings suggest that nutritional screening in ED patients with DM and/or CKD may help identify a subgroup with greater vulnerability, but screening results should not be used in isolation to guide admission decisions. Instead, nutritional risk should be interpreted together with renal function, inflammatory markers, acute diagnosis, functional status, comorbid disease burden, and clinical judgment. The moderate descriptive separation observed for NRS-2002 and MNA also supports this cautious interpretation: these tools may contribute useful information, but they should not be considered sufficient for disposition decisions in isolation. In discharged patients, recognition of nutritional vulnerability may still be clinically relevant because these patients may benefit from outpatient follow-up, dietary counseling, or further assessment after the acute ED encounter.

### 4.1. Limitations

This study has several limitations. First, the single-center design and small sample size limit generalizability and restrict the number of covariates that can be included in multivariable models. In addition, the exploratory logistic regression model was fitted at the lower boundary of recommended stability, with 51 admission events for five conceptual predictors. Because MNA nutritional status was modeled categorically, the effective number of predictor parameters was higher, further reducing the events-per-parameter ratio. The wide confidence interval for MNA-defined malnutrition and the limited number of patients in the malnourished category indicate that the adjusted estimates should be interpreted cautiously. We also did not perform a parallel adjusted model substituting NRS-2002 for MNA because NRS-2002 includes a disease-severity component that overlaps conceptually with CRP and serum urea; therefore, such a model would have been difficult to interpret and potentially vulnerable to overadjustment in this small cohort. Second, there is substantial potential for selection bias, confounding by indication, and possible reverse causation because group allocation was based on the clinical disposition decision after ED evaluation. Clinicians’ admission decisions may have been influenced by overall appearance, frailty, reduced oral intake, dehydration, functional decline, and perceived physiological reserve, which overlap with the constructs assessed by NRS-2002 and MNA. Therefore, the observed association between nutritional risk and admission should not be interpreted as evidence that nutritional status independently determines disposition. These groups likely also differed in acute illness severity, renal dysfunction, inflammatory burden, and clinician disposition practices. Third, nutritional assessments were performed during the ED encounter and around the time of the disposition decision, which precludes strong inference about prospective prediction before disposition. Fourth, although adjustment was performed for available variables, residual confounding may remain because detailed disease-specific variables such as eGFR, CKD stage, dialysis status, diabetes duration, HbA1c, infection severity, functional status, medication use, and comorbidities were not available for inclusion in the adjusted analyses. Because eGFR, CKD stage, and dialysis status were not systematically available in the dataset, serum urea and creatinine could only be interpreted as crude markers of renal and metabolic severity rather than as precise indicators of CKD stage. In particular, chronic inflammatory or immune-mediated diseases were not systematically recorded, and residual confounding related to inflammatory burden may have remained despite the inclusion of CRP as an available inflammatory marker. Fifth, body weight and height were obtained from patient self-report or from existing medical records when direct measurement was not feasible; therefore, recall bias and measurement error may have affected BMI values and anthropometric subgroup analyses. Sixth, the study did not assess whether nutritional interventions altered clinical outcomes, subsequent healthcare utilization, or post-discharge trajectories. Seventh, length of stay, mortality, and readmission data were not available. Eighth, the MNA was originally developed and validated mainly for older adults; therefore, its use in a broader adult ED population, including 33 patients younger than 65 years, may limit the generalizability and interpretation of MNA-based findings. Although we added a descriptive age-stratified comparison of MNA categories, this analysis was underpowered and did not allow reliable assessment of age-specific MNA performance or an age-restricted adjusted model. Future studies should either restrict MNA analyses to older adults or perform adequately powered age-stratified analyses.

### 4.2. Future Directions

Future research should focus on multicenter studies with larger sample sizes to confirm these findings. Longitudinal studies with standardized pre-disposition nutritional assessment are needed to evaluate whether nutritional risk is independently associated with admission decisions and subsequent outcomes such as length of stay, readmission, mortality, and functional decline. Future interventional studies may also clarify whether early nutritional assessment and targeted follow-up can improve clinical outcomes in patients with DM and/or CKD.

## 5. Conclusions

In this exploratory single-center study, nutritional risk was more common among admitted ED patients with DM and/or CKD than among discharged patients. However, this association appeared to overlap with markers of renal and metabolic severity, particularly serum urea. Nutritional screening, particularly using adult-appropriate tools such as NRS-2002 and complementary assessment with MNA in older patients, may help identify clinically vulnerable patients in the ED. However, larger prospective studies with standardized pre-disposition assessment, age-stratified nutritional analyses, and adjustment for disease severity are needed before nutritional scores can be considered independent markers of admission status or incorporated into disposition-related risk models.

## Figures and Tables

**Table 1:** Nutritional risk and malnutrition status by sex.

Nutritional Classification		Gender				Sum	
		F (n = 48)		M (n = 53)			
		n	%	n	%	n	*p* *
Nutritional risk according to NRS-2002	No nutritional risk	22	45.8%	31	58.5%	53	0.233
	Nutritional risk present	26	54.2%	22	41.5%	48	
Malnutrition status according to MNA score	Normal	5	10.4%	16	30.2%	21	0.014
	There is a risk of malnutrition	24	50.0%	27	50.9%	51	
	There is malnutrition	19	39.6%	10	18.9%	29	

*: Pearson chi-square test. Percentages are calculated within each sex category.

**Table 2:** Comparison of blood samples taken from admitted and discharged groups.

Parameter	Group	n	Mean	SD	Percentiles			*p*
					25th	Median	75th	
Hemoglobin * (g/dL)	Admitted group	51	11.16	2.92	9.40	11.30	12.50	0.705
	Discharged group	50	11.37	2.44	9.07	11.40	13.15	
Hematocrit * (%)	Admitted group	51	34.33	8.84	28.00	35.00	38.00	0.778
	Discharged group	50	34.92	6.65	28.75	35.00	40.00	
Lymphocyte (10^3/uL)	Admitted group	51	1.28	0.940	0.60	1.10	1.60	0.001
	Discharged group	50	1.69	1.09	1.00	1.35	2.00	
CRP (mg/L)	Admitted group	50	93.90	107.57	12.50	52.00	139.75	0.001
	Discharged group	50	26.53	35.50	2.18	12.88	30.50	
Glucose (mg/dL)	Admitted group	51	240.51	213.67	109.00	143.00	280.00	0.108
	Discharged group	50	176.08	133.14	101.00	122.00	213.00	
Urea (mg/dL)	Admitted group	51	138.06	83.67	81.00	124.00	183.00	0.001
	Discharged group	50	87.82	50.11	51.25	77.50	115.50	
Creatinine (mg/dL)	Admitted group	51	3.99	2.84	1.93	2.58	3.73	0.049
	Discharged group	50	3.18	2.99	1.27	1.66	4.01	
Calcium (mg/dL)	Admitted group	51	9.17	1.70	8.50	8.80	9.40	0.106
	Discharged group	50	9.14	0.78	8.67	9.15	9.65	
Albumin (g/L)	Admitted group	51	36.43	10.52	31.30	36.20	42.00	0.085
	Discharged group	50	38.68	7.13	34.80	39.05	43.53	
Protein * (g/L)	Admitted group	50	66.49	12.33	61.75	68.55	74.00	0.167
	Discharged group	50	69.39	8.05	64.45	70.25	74.30	

*: Independent samples *t*-test was used for normally distributed variables marked with an asterisk. Mann–Whitney U test was used for all unmarked variables, including lymphocyte count, CRP, glucose, urea, creatinine, calcium, and albumin.

**Table 3:** Distribution of MNA categories according to age group.

MNA Category	<65 Years (n)	<65 Years (%)	≥65 Years (n)	≥65 Years (%)	Total (n)
Normal	11	33.3%	10	14.7%	21
At risk of malnutrition	15	45.5%	36	52.9%	51
Malnourished	7	21.2%	22	32.4%	29
Total	33	—	68	—	101

**Table 4:** Comparison of patients with and without nutritional risk according to NRS-2002 in the admitted and discharged groups.

Group		Nutritional Risk According to NRS-2002	n	Mean	SD	*p*
Admitted group	Age	No nutritional risk	18	48.11	19.64	0.001
		Nutritional risk	33	77.09	10.98	
	Height	No nutritional risk	18	169.44	9.00	0.157
		Nutritional risk	33	166.67	9.59	
	Weight *	No nutritional risk	18	76.89	20.36	0.235
		Nutritional risk	33	71.03	14.27	
	BMI	No nutritional risk	18	26.59	6.10	0.574
		Nutritional risk	33	25.63	5.44	
Discharged group	Age	No nutritional risk	35	61.86	17.52	0.001
		Nutritional risk	15	80.07	10.07	
	Height	No nutritional risk	35	172.00	7.89	0.008
		Nutritional risk	15	165.20	8.58	
	Weight *	No nutritional risk	35	76.20	11.32	0.016
		Nutritional risk	15	68.53	5.15	
	BMI	No nutritional risk	35	25.72	3.22	0.597
		Nutritional risk	15	25.22	2.43	

*: The independent samples *t*-test was used to compare the two groups in terms of normally distributed traits. The Mann–Whitney U test was used for comparison in terms of other characteristics.

**Table 5:** Comparison of anthropometric variables across MNA nutritional categories in the admitted and discharged groups.

Group	Malnutrition Status According to MNA Score		n	Mean	SD	Percentiles			*p*
						25th	Median	75th	
Admitted group	Age	Normal	6	57.83	19.94	42.75	56.00	77.00	0.121
		There is a risk of malnutrition	22	62.91	21.05	45.25	69.00	78.25	
		There is malnutrition	23	73.00	18.10	64.00	76.00	85.00	
	Height	Normal	6	177.67	2.94	176.50	178.00	180.00	0.003
		There is a risk of malnutrition	22	168.50	8.50	162.00	169.00	176.00	
		There is malnutrition	23	164.22	9.43	158.00	162.00	175.00	
	Weight *	Normal	6	90.50	16.36	80.00	85.00	98.25	0.001
		There is a risk of malnutrition	22	77.50	14.78	67.25	75.00	90.00	
		There is malnutrition	23	64.35	13.54	52.00	65.00	72.00	
	BMI	Normal	6	28.66	5.05	25.99	26.93	30.54	0.010
		There is a risk of malnutrition	22	27.40	5.73	24.04	25.61	28.65	
		There is malnutrition	23	23.91	5.17	20.03	23.44	25.97	
Discharged group	Age	Normal	15	60.60	18.47	53.00	64.00	71.00	0.090
		There is a risk of malnutrition	29	69.31	15.92	62.50	72.00	81.00	
		There is malnutrition	6	74.50	21.77	59.75	78.50	93.25	
	Height	Normal	15	174.20	6.19	167.00	176.00	178.00	0.057
		There is a risk of malnutrition	29	168.55	9.03	162.00	172.00	176.00	
		There is malnutrition	6	166.17	8.95	159.50	162.00	176.75	
	Weight *	Normal	15	78.67	8.10	75.00	75.00	83.00	0.007
		There is a risk of malnutrition	29	73.62	10.57	65.00	72.00	80.00	
		There is malnutrition	6	63.33	7.53	57.50	65.00	70.00	
	BMI	Normal	15	25.92	2.23	24.21	25.83	26.89	0.137
		There is a risk of malnutrition	29	25.92	3.17	23.53	25.59	28.11	
		There is malnutrition	6	23.00	2.87	20.50	22.94	25.71	

*: One-way ANOVA was used to compare more than two groups for normally distributed traits. The Kruskal–Wallis test was used for comparison in terms of other characteristics.

**Table 6:** Distribution of wards where admitted patients are inpatients.

Group		n	%
Admitted group	Internal Medicine—Acute Kidney Injury	36	70.6
	Internal Medicine—Acute Kidney Injury + Hypernatremia	1	2.0
	Internal Medicine—Diabetic Ketoacidosis	8	15.7
	Internal Medicine—Blood Gas Test Regulation	1	2.0
	INTENSIVE CARE UNIT—Acute Kidney Injury	3	5.9
	ICU—Acute Kidney Injury + Diabetic Ketoacidosis	2	3.9
	Sum	51	100.0

**Table 7:** Exploratory univariable ROC analyses for hospital admission.

Risk Factor	AUC	*p* Value	95% CI Lower	95% CI Upper
NRS-2002	0.733	<0.001	0.631	0.835
MNA	0.722	<0.001	0.622	0.822
CRP	0.726	<0.001	0.627	0.825
Urea	0.709	<0.001	0.608	0.810
Creatinine	0.619	0.041	0.505	0.733
Age	0.530	0.608	0.415	0.644
Lymphocyte count	0.820	<0.001	0.721	0.920

**Table 8:** Exploratory multivariable logistic regression model for hospital admission.

Variable	*p* Value	OR	95% CI Lower	95% CI Upper
Age	0.062	0.971	0.942	1.002
Sex (Female/Male)	0.043	0.307	0.098	0.965
C-reactive protein	0.002	1.022	1.008	1.036
Urea	0.001	1.018	1.007	1.029
MNA-defined malnutrition status	0.022	—	—	—
MNA status (At risk/Normal)	0.523	1.601	0.378	6.779
MNA status (Malnourished/Normal)	0.016	7.926	1.482	42.391
Constant	0.292	0.330	—	—

## Data Availability

The data supporting the findings of this study are available from the corresponding author upon reasonable request.

## Abbreviations

The following abbreviations are used in this manuscript:

AKI	Acute kidney injury
AUC	Area under the curve
BMI	Body mass index
CI	Confidence interval
CKD	Chronic kidney disease
CRP	C-reactive protein
DM	Diabetes mellitus
ED	Emergency department
HbA1c	Hemoglobin A1c
ICU	Intensive care unit
KDIGO	Kidney Disease: Improving Global Outcomes
MNA	Mini Nutritional Assessment
NRS-2002	Nutritional Risk Screening-2002
OR	Odds ratio
ROC	Receiver operating characteristic
SD	Standard deviation
SPSS	Statistical Package for the Social Sciences
STROBE	Strengthening the Reporting of Observational Studies in Epidemiology
